# Development of Traumatic Brain Injury Associated Intracranial Hypertension Prediction Algorithms: A Narrative Review

**DOI:** 10.1089/neu.2022.0201

**Published:** 2023-03-01

**Authors:** Robert McNamara, Shiv Meka, James Anstey, Daniel Fatovich, Luke Haseler, Toby Jeffcote, Andrew Udy, Rinaldo Bellomo, Melinda Fitzgerald

**Affiliations:** ^1^ Department of Intensive Care Medicine, Royal Perth Hospital, Perth, Western Australia, Australia.; ^2^ School of Medicine, Curtin University, Bentley, Western Australia, Australia.; ^3^ Data Innovation Laboratory, Western Australian Department of Health, Perth, Western Australia, Australia.; ^4^ Department of Intensive Care, The Royal Melbourne Hospital, Melbourne, Victoria, Australia.; ^5^ Department of Emergency Medicine, Royal Perth Hospital, Perth, Western Australia, Australia.; ^6^ Centre for Clinical Research in Emergency Medicine, Harry Perkins Institute of Medical Research, Perth, Western Australia, Australia.; ^7^ Curtin Health Innovation Research Institute, Curtin University, Bentley, Western Australia, Australia.; ^8^ Department of Intensive Care, Alfred Health, Melbourne, Victoria, Australia.; ^9^ Australian and New Zealand Intensive Care Research Centre, School of Public Health and Preventive Medicine, Monash University, Melbourne, Australia.; ^10^ Department of Critical Care, The University of Melbourne, Melbourne, Victoria, Australia.; ^11^ Department of Intensive Care, Austin Hospital, Melbourne, Australia; ^12^ Data Analytics Research and Evaluation, Austin Hospital, Melbourne, Australia; ^13^ Perron Institute for Neurological and Translational Sciences, Nedlands, Western Australia, Australia.

**Keywords:** intracranial hypertension, intracranial hypertension prediction, intracranial pressure forecasting, machine learning

## Abstract

Traumatic intracranial hypertension (tIH) is a common and potentially lethal complication of moderate to severe traumatic brain injury (m-sTBI). It often develops with little warning and is managed reactively with the tiered application of intracranial pressure (ICP)-lowering interventions administered in response to an ICP rising above a set threshold. For over 45 years, a variety of research groups have worked toward the development of technology to allow for the preemptive management of tIH in the hope of improving patient outcomes. In 2022, the first operationalizable tIH prediction system became a reality. With such a system, ICP lowering interventions could be administered prior to the rise in ICP, thus protecting the patient from potentially damaging tIH episodes and limiting the overall ICP burden experienced. In this review, we discuss related approaches to ICP forecasting and IH prediction algorithms, which collectively provide the foundation for the successful development of an operational tIH prediction system. We also discuss operationalization and the statistical assessment of tIH algorithms. This review will be of relevance to clinicians and researchers interested in development of this technology as well as those with a general interest in the bedside application of machine learning (ML) technology.

## Introduction

One of the most common complications of moderate to severe traumatic brain injury (m-sTBI) is a rise in intracranial pressure (ICP).^1,2^ If significant, rises in ICP cause traumatic intracranial hypertension (tIH), which promotes further damage to the injured brain^3,4^ and is strongly associated with poor outcomes.^5–7^ Accordingly, a cornerstone of m-sTBI intensive care unit (ICU) neuroprotective management is the monitoring of ICP and treatment of tIH using a strategy that escalates in intensity in response to ICP rises.^2,8–10^ Due to the pathophysiology of tIH, wherein rapid ICP rises occur as intracranial capacity is reached, the treatment of tIH is universally reactive. Despite numerous observational trials demonstrating benefit of ICP-guided m-sTBI treatment algorithms,^11^ multiple interventional trials aimed at reducing ICP values have failed to demonstrate benefit in patient outcomes.^12–17^ One potential explanation for this apparent lack of benefit is the reactive application of the interventions tested so far, whereby treatments are administered after damaging tIH episodes have become established.^12,14^ This has led many to theorize that preempting ICP rises will offer a better approach and lead to improved outcomes.

## Pathophysiology of Intracranial Hypertension

With multiple linear and non-linear related determinants, ICP is a highly complex parameter, which is influenced by many extra- and intracranial factors.^2,3,10,18–20^ ICP is most simply conceptualized as being determined by the volume of the classical brain compartments contained by the skull: blood, cerebral spinal fluid, and brain tissue.^3,21^ In sTBI and some other conditions, there is the additional fourth compartment of extra-axial blood.^3,21^ Less common non-traumatic causes of IH include hemorrhagic stroke, subarachnoid hemorrhage, central venous thrombosis, acute hydrocephalus, meningitis, encephalitis, brain abscesses, tumors, and metabolic insults such as hyperammonemia and ischemic/hypoxic brain injury. In tIH, there is a gradual rise in pressure as the contained volume increases. Additional volume beyond capacity leads to an exponential rise in pressure. At maximum cranial capacity, a small further increase in volume rapidly results in a large increase in pressure. This is the fundamental reason for the rapid rises in ICP frequently experienced by patients with m-sTBI—the volumes inside the skull increase beyond the skull's capacity and trigger a rapid rise in ICP.^21^ In the setting of m-sTBI, expansion of the blood, extra-axial blood and/or brain tissue compartments due to loss of autoregulation, bleeding, and/or swelling of brain tissue, respectively, are the most common causes of tIH.^11^

IH can be damaging via two mechanisms. Firstly, the direct pressure effect causes displacement or herniation of brain tissue, resulting in further damage to the injured brain due to direct neuronal injury in addition to disruption of insulating myelin and other supporting glia.^4,21,22^ A cascade of biochemical events is triggered, leading to inflammation that is often associated with disruption of local blood supply.^4,21^ The second mechanism results from the elevated ICP impeding the blood flow to the brain resulting in a global reduction in brain perfusion.

In mild to moderate cases of tIH, this impedance, in combination with several other factors, contributes to diffuse ischemic injury, which commonly occurs in severe TBI.^22–24^ In severe cases of IH, this impedance results in death due to complete cessation of blood flow to the brain.^4,21,22^ IH events are the culmination of several neuropathophysiological processes and are both a consequence and a cause of secondary brain injury.^2,10,18,25^ As such, IH events are strongly predictive of further IH events.^26–29^ Significant tIH events, wherein the ICP rise above 22 mm Hg, are unfortunately common following m-sTBI.^2,30,31^ Local data demonstrate that, on average, each patient has three to four of these potentially damaging episodes lasting ≥5min during the period of neuromonitoring, in spite of guideline-based ICU management and continuous monitoring.^1,8,9^ There is a substantial body of evidence linking tIH with poor outcomes in patients with m-sTBI, particularly in the setting of impaired cerebral autoregulation.^5,7–9,26,31–36^ Despite this, the treatment of tIH remains reactive in current practice.^8,9^

## Forecasting and Prediction Algorithms

Our final goal is to develop an expert bedside system that can automatically analyse versatile information about the patient and recognise, as early as possible, those combinations of symptoms that correspond to a state of emergency.^37^

The concept of developing an early warning system for tIH is not new. It has long been known that prevention of a disease or early detection and intervention into a disease process can lead to better outcomes. This maxim of medicine has led to the hypothesis that prevention and/or early intervention into episodes of tIH will lead to improved outcomes. Several groups have attempted to develop the ability to forecast ICP values and/or predict tIH episodes over the past 45 years. Common to all algorithms developed to date is a predominant reliance on ICP wave analysis for prediction.

These efforts can be broadly classified into two related but distinct approaches: 1) ICP forecasting, which involves the development of algorithms designed to predict all future ICP values, and 2) tIH prediction, which involves the development of algorithms designed to specifically provide warning of an impending tIH event. Despite steady advances in both ICP forecasting and tIH prediction algorithm development, these, until very recently,^29^ have been limited to the analysis of retrospective data. Several factors have been limiting the translation of these algorithms into clinical practice, including the generalizability-limiting heuristic nature of their development; high feature and/or pre-processing requirements, which results in computational demands that preclude real-time signal processing; and/or the need for a prolonged period of data collection prior to first prediction.

## Search Method

The following databases were examined to undertake this literature review: MEDLINE (1966 to present), Embase (1980 to present), and Google Scholar. The search strategy used the following Medical Subject Headings (MeSH) terms: intracranial hypertension forecasting, intracranial hypertension prediction, intracranial pressure prediction, intracranial pressure forecasting, intensive care. All MeSH terms were exploded. Articles were retrieved and examined for relevance to the subject. In addition, references of identified articles were searched and relevant articles retrieved.

## Early Efforts

The earliest studies into the development of ICP forecasting and tIH prediction algorithms occurred in the 1970s and applied histogram and linear statistical approaches to the analysis of the ICP waveform.^38–43^ In 1976, Turner and colleagues described a “simple analysis of ICP as an aid for the prediction of a rise in ICP.”^39^ In this method, the investigators recorded ICP values every 4 sec for 33 min and, from this, compiled a histogram to display the pressure frequency distribution.^39^ They found that increases in ICP histogram rightward skew portended a rise in ICP, and, in three patients, found that ICP pulse pressure increased in the period immediately prior to an ICP rise. Variation due to cardiac impulses and/or the respiratory cycle in addition to random variation were noted to limit the application of this technique.^39^

Later, in 1980, Price and associates described automation of a three-stage ICP management algorithm to detect and treat tIH. In this process, the ICP pulse wave mean (PWM) was divided by the ICP pulse wave amplitude (PWA) to generate the pulse wave index (PWI).^40^ In cases of a rise in the PWI due to a fall in PWA, furosemide was administered. In cases of an increase in the PWI due to an increase in PWM, mannitol was administered.^40^ In the final control loop of the algorithm, in the event cerebral perfusion pressure (CPP) fell below 50 mm Hg, external ventricular drain venting was initiated. Using this approach for early detection of tIH, the investigators noted that unit mortality fell from 51% to 36%, whereas survivors with severe disability fell from 18% to 3%. They concluded that by using the three decoupled intervention loops, “control of intracranial hypertension is established with greater ease and precision.”^40^

In terms of tIH prediction, although the use of histograms and other linear statistical models facilitated early recognition of an ICP rise, the process required ICP rises to already begin occurring and thus were not true methods of tIH prediction, but rather an attempt at earlier recognition of, and intervention for, a tIH event. In 1983, Allen published a two-part series describing a variety of time-series linear statistical approaches that theoretically could be used for analysis of the ICP wave and for the potential provision of a tIH early warning.^41^ He noted that the development of such a system “demands close cooperation between the clinician and the engineer in order to define realistic objectives and achieve satisfactory implementation.”^41^ In his first article, Allen described a number of control system engineering-derived time-series analytical methods that could theoretically be applied to the problem of ICP forecasting.^41^ These methods included the use of Box-Jenkins models (a family of linear, univariate, and stochastic models developed by Box and Jenkins in 1970 and 1979, respectively) combined with autoregressive-integrated moving average (ARIMA) modeling, as well as the application of Kalman filtering algorithms.^41^ Other methods described include the recursive Holt algorithm, as well as the Holt algorithm's proposed extensions by Trigg and Leach.^41^

In the second part, Allen described the preliminary use of such systems using retrospective data.^42^ He found that the Box-Jenkins ARIMA modeling and forecasting was unsuitable to the task of ICP forecasting. However, he noted that it was “a valuable method for investigating the characteristics of the data.” He concluded that “the most promising method studied was the use of a dynamic linear model with a recursive parameter estimator in an adaptive framework.”^42^ These early efforts demonstrate that the concept of earlier intervention and/or prevention of ICP rises was present from the beginning of computer analysis of ICP data. Major barriers to the development of ICP forecasting and/or tIH algorithms at that time included the need for processing multiple linear and non-linear parameters, which was precluded by the inadequate computing technology and statistical methodology of the time. Accordingly, it wasn't until the advent of machine learning (ML) methodologies, better suited for the analysis of multiple non-linear relationships, and better computing technology in the 1990s that the computational power and analytical methodology capable of handling the computational burden became available.

## ICP Forecasting Algorithms

Since the mid-1990s there have been several attempts to develop ICP forecasting algorithms (Table 1). The earliest published record of an attempt to develop an ICP forecasting algorithm using ML techniques was by Swiercz and co-workers in 1995.^44^ In this approach, analysis of mean ICP waveform data using an artificial neural network (ANN) was undertaken to identify features that could provide clinicians with a forecast of future ICP values and alert them of an impending tIH event. However, although successful, the warning time horizon of <5 sec was too short to be clinically useful and the method could only be performed retrospectively due to computational requirements and a need to remove artefacts prior to processing.^44^

**Table 1.** Summary of ICP Forecasting Studies

Research group	Signals	Sampling frequency	Pre-processing	Analysis	Results	Time horizon
Swiercz, et al. (1995)	ICP	51 Hz	1 sec average values generated with subsequent averaging into 30-sec sliding windows, spectral analysis, logarithmic normalization of ICP signal	ANN	Proof of concept work to demonstrate ability of an ANN to forecast ICP values 5 min in advance; graphical data only; no numerical data provided	5 min
Tsui, et al. (1995)	ICP	200 Hz	DWT	RNN	Mean error 4.51% on initial prediction processing	32 and 64 sec
Swiercz, et al. (1998)	ICP	51 Hz	Segmentation, spectral analysis + digital filtering, calculation of ICP second order statistics and RAP; computation of non-stationarity indices of ICP wave	ANN + spectral analysis	ARV 0.806	3 min
Swiercz, et al. (2000)	ICP	51 Hz	10-sec timestep segmentation	1) ANN with non-linear AR; 2) ANN with wavelet decomposition analysis; 3) classical AR with Kalman filter	1) ANN with non-linear AR analysis: MAE <3.72%; 2) ANN with wavelet decomposition: MAE <3.21%; 3) Kalman filter with AR: ARV 0.75	3 min
Shieh, et al. (2004)	ICP, MAP, ECG, EtCO_2_, rSO_2_	0.25 Hz	Median filter algorithm applied	SRNNTT	Mean error 1.8%	N/A
Zhang, et al. (2011)	ICP, MAP, P_bt_O_2_, brain temperature	100 Hz	Artefact removal, missing data cleaning, data segmentation and extraction of segment means	ANN_NARX_-MFA	ANN_NARX_-MFA T = 15 min: RAE 9% ± 3% , CoD 0.93 ± 0.05; T = 30 min: RAE 24% ± 11%, CoD 0.81 ± 0.11	15, 30, and 45 min
Feng, et al. (2012)	ICP, MAP, P_bt_O_2_, PRx	0.2 Hz	Segmentation; artefact and ICP intervention removal; linear regression of segments; discretization of regression analysis into "elevate," "stay," or "reduce"	1) AODE; 2) AdaBoost-J48; 3) BayesNet-K2/TAN); 4) LBR; 5) LogReg; 6) Naive Bayes; and 7) SVM	Temporal approach average: accuracy 62.2%, AUROC 0.641, F-Measure 62.2%; non-temporal approach average: accuracy 53.3%, AUROC 0.533, F-Measure 52.9%	60 min
Han, et al. (2013)	ICP	0.1 Hz	Outlier removal, empirical mode decomposition, artefact removal	ARIMA + VS; ARIMA + RW	ARIMA + VS: ODA 99.62%, MSRE 3.22%, GPER 4.19%; ARIMA + RW: ODA 99.58%, MSRE 3.13, GPER 3.53%;	60 min
Bonds, et al. (2015)	ICP, HR, SBP, MAP, SI, PP	0.167 Hz	5-min moving window, continuous averaging of window waveforms	NNR	NNR prediction of ICP: @ 5 min: bias of 0.02 (T2 SD = 4 mm Hg); @ 2 h: bias of 0.02 (T2 SD = 10 mm Hg)	5 min, 2 h
Farhadi, et al. (2018)	ICP, MAP, CPP, BP, RR, HR	0.167 Hz	Imputed missing data if <50% in data set; if >50%, data segment deleted	ARIMA and exponential smoothing models; random forest method	Random forest model RMSE 0.89%; correlation between ICP forecast and experimental values 0.99	30 min
Ye, et al. (2022)	ICP	50 HZ	Denoising, smoothing, and outlier removal; forward imputation of missing data	RNN - LSTM	Average accuracy, sensitivity, specificity, and RMSE: 94.62%, 74.91%, 94.83%, and 2.18 mm Hg	10 min

AdaBoost-J48, Ada-Boosting with decision tree; ANN, artificial neural network; ANNNARX-MFA, non-linear autoregression with exogenous input artificial neural network based mean forecast algorithm; AODE, aggregating one-dependence estimators; AR, autoregression; ARIMA, autoregression integrated moving average; ARV, average relative variance coefficient; AUROC, area under the receiver operating characteristic; BayesNet-K2/TAN, Bayesian network with K2 & TAN; BP, blood pressure; brain temp, brain temperature; CoD, coefficient of determination; CPP, cerebral perfusion pressure; DWT, diffuse wavelet transformation; ECG, electrocardiogram; ETCO_2_, end-tidal carbon dioxide; GPER, gross prediction error rate; HR, heart rate; ICP, intracranial pressure; LBR, lazy Bayesian rules; LogReg, logistic regression; LSTM, long short-term memory; MAE, maximum absolute error; MAP, mean arterial pressure; MSRE, mean signal reconstruction error; Naive Bayes, naïve Bayesian classifier; NNR, nearest neighbor regression; ODA, outlier detection accuracy; P_bt_O_2_, brain tissue oxygenation; PRx, pressure reactivity index; RAE, relative absolute error; RAP, index of compensatory reserve; RMSE, root mean square error; RNN, recurrent neural network; RR, respiratory rate; rSO_2_, regional oxygenation saturation; RW, random walk model; SRNNTT, simple recurrent neural network structure through time; SVM, support vector machine; VS, velocity sensor model.

In the same year, as part of doctoral work examining the use of wavelets in time-series prediction, Tsui and colleagues presented work describing the application of ICP waveform discrete wavelet transformation (DWT) pre-processing prior to analysis by a recurrent neural network (RNN).^45^ This method demonstrated a high degree of accuracy for time horizons of 32 and 64 sec prior to event and required approximately 20 min of data prior to being able to begin forecasting future ICPs. During thesis work on the development of time-series data forecasting models, Tsui presented several additional studies involving the use of wavelet pre-processing of TBI patient ICP wave data prior to ML algorithm processing.^46,47^ This work is described in his doctoral thesis.^48^ Swiercz's group subsequently published DWT pre-processing of 1-h ICP waveform traces prior to ANN analysis. In this later effort, Swiercz's group also incorporated other values (SpO_2_, mean arterial pressure [MAP], etc.) into their algorithm.^37^ Using this method, they were able to forecast ICP values 3 min in advance with a high degree of accuracy.^37^ However, neither group was able to operationalize the DWT pre-processing with an ANN or RNN analysis approach due to the high computational requirements and the need for stable and artefact-free ICP data.

In 2011, Zhang and associates^49^ described a non-linear autoregressive with exogenous input artificial neural network-based mean forecast algorithm (ANN_NARX_-MFA) to predict ICP. This algorithm, which extracted features from past windows and segmented sub-windows for processing, was shown to be superior (15-min coefficient of determination [R^2^] 0.93 ± 0.05 and relative absolute error [RAE] 9% ± 3%) to an ANN_NAR_ algorithm (15-min R^2^ 0.88 ± 0.07 and RAE 15% ± 5%), which did not incorporate feature extraction.^49^ In a 2012 follow-up study by the same group, Feng and co-workers demonstrated that a forecasting model utilizing temporal information on historical ICP readings and related parameters (MAP, pressure reactivity index [PRx], and brain tissue oxygenation [P_bt_O_2_]) improved forecasting performance by an average of 20% (*p*-value <0.001) compared with algorithms that did not make use of such information.^50^

In 2013, Han and colleagues presented results of their group's ARIMA-based ICP forecasting approach, which included an online algorithm capable of processing clinically derived data captured at a low rate (0.1 Hz) of signal sampling.^51^ In this effort, non-stationary ICP data and movement artefacts were removed using empirical mode decompression (EMD) and robust estimation pre-processing.^51^ Simulation testing of the approach demonstrated good performance, with a gross prediction error rate of <5% using robust updating and smoothing pre-processing prior to ARIMA analysis. Further efforts at ICP forecasting were published in 2015 when Bonds and associates described their method of using nearest neighbor regression (NNR) analysis to forecast ICP.^52^ In this model, patient monitoring data captured every 6 sec from 132 adult patients with m-sTBI were used to generate short trends of vital sign data (systolic blood pressure [SBP], MAP, ICP, heart rate, shock index, and pulse pressure). Using these short trend segments, a series of vital sign patterns with a corresponding ICP was generated. These patterns were then used to predict ICP by assigning the ICP value linked with a known vital sign pattern to those with similar vital sign patterns (i.e., the nearest neighbor).^52^ Using this NNR method, the group demonstrated the ability to predict ICP values with a bias of 0.02 for the 5-min horizons (± 2 standard deviation [SD] = 4 mm Hg) and −0.02 for the 2-h horizon (± 2 SD = 10 mm Hg).^52^

In 2019, Farhadi and co-workers described the use of ARIMA and exponential smoothing (ETS) models in addition to random decision tree ML statistics to forecast ICP in children with m-sTBI.^53^ This method demonstrated a root mean square error (RMSE) of 0.89% when medications administered within the previous 2 h were incorporated. Further work using RNN analysis is noted to be planned by the Farhadi group.^53^ In 2022, Ye and colleagues published results of a RNN long short-term memory (LSTM) algorithm trained to forecast ICP values with a 10-min horizon.^54^ The algorithm was trained using 50 Hz ICP wave data from 13 patients with TBI. Overall the LSTM model average accuracy, sensitivity, specificity, and RMSE was noted to be 94.62%, 74.91%, 94.83%, and 2.18 mm Hg, respectively.^54^

## tIH Prediction Algorithms

More recently, there has been a greater focus on the development of tIH prediction algorithms that provide an early warning of an impending tIH event (Table 2). Current guidelines define tIH as an ICP value of ≥22 mm Hg lasting for ≥5 min.^8,9^ Prior to 2018, the definition of tIH was an ICP of ≥20 mm Hg lasting for ≥5 min.^55^ The tIH event definition used for algorithm event detection varies among research groups, with some groups using the pre-2018 ICP threshold.^56–58^ Other definitions include ICP >30 mm Hg for >10 min,^59^ ICP >25 mm Hg for >5 min^60^ and ICP >20 mm Hg for >15 min.^26^ From a technical perspective, the ease of algorithm development is inversely related to the severity of the tIH definition used. In other words, providing sufficient tIH event data are present in the training data, the longer and higher the ICP threshold used to define a tIH event is, the easier it is to develop an algorithm. Regardless of the definition used, tIH prediction algorithms can be broadly classified into two approaches based on the methodology used to predict tIH: ICP morphology-based or ML-based approaches.

**Table 2.** Summary of tIH Prediction Studies

Research group	Signals	Sampling frequency	Pre-processing	Analysis methods	tIH definition	Comment	Main results	Time horizon
Mariak, et al. (2000)	ICP	51 Hz	Spectral analysis, digital filtering, second order analysis of ICP values, computation of RAP, non-stationarity indices of the ICP signal	ANN (predictive modular neural network)	N/A	ANN algorithm trained to classify ICP waveforms into 1 of 4 expert-determined categories	70% correlation with expert opinion	N/A
Hu, et al. (2009)	ICP, ECG	240–400 Hz	Segmentation of wave data preceding ICP rises	Hierarchical clustering and pulse analysis using reference library of 10 non-artefactual dominant ICP pulses	N/A	MOCAIP proof-of-concept article	Accuracy of 90.17%, 87.56%, and 86.53% for designating each established ICP sub-peak	N/A
Fan, et al. (2010)	ICP, ABP	5-sec average	Minutely averaged, artefact and outlier removal, forward and backward imputation if required	Standard statistical analysis: mean, slope, ANOVA, standard deviation, variance	Increase in ICP >10 mm Hg over baseline for >5 min if the baseline <20 mm Hg, or ICP >25 mm Hg for >5 min if baseline >20 mm Hg	Calculated slope of the ICP waveform in 5–20, 10–20, 20–30 min windows prior to the ICP rise; authors noted that this technique was not clinically viable	Decrease in slope in 10–20 min vs. the 20–30 min window (MD = 0.752, *p* = 0.007); 5–20 min window vs. 20–30 min window (MD = 0.769, *p* = 0.049)	5–30 min
Hu, et al. (2010)	ICP, ECG	240–400 Hz	Quadratic classifier and ICP and ECG segments extracted for periods from 20 min before to 1 min after the onset of ICH event; clustering of average representative pulse from 1-min segments	Differential evolution ML algorithm + MOCAIP; hierarchical clustering and pulse analysis using reference library of 10 non-artefactual dominant ICP pulses	>20 mm Hg for >5 min	MOCAIP validation study; no patients with TBI included	5 min: sens 0.78, spec 0.97, acc 0.917 ± 0.005; 20 min: sens 0.723 ± 0.006; spec 0.721 ± 0.003; acc 0.721 ± 0.002	5 min and 20 min
Scalzo, et al. (2012)	ICP, ECG	240 or 400 Hz	ICP and ECG segments extracted to cover period from 20 min before to 1 min after onset of ICH event; clustering of average representative pulse from 1-min segments; segment analysis	Morphology analysis and supervised ML: multiple linear regression, adaptive boosting, extremely randomized decision trees	>20 mm Hg for >5 min	MOCAIP refinement study; no patients with TBI included	Full feature MOCAIP AUROC @ 1 min: 0.76; AUROC @ 3 min: 0.66; AUROC @ 6 min: 0.58; results using “best” 13 MOCAIP metrics: AUROC @ 1 min: 0.87; AUROC @ 3 min: 0.78; AUROC @ 6 min: 0.71	1, 3, and 6 min
Guiza, et al. (2013)	ICP, ABP	1-min average	No	GP	>30 mm Hg for >10 min	Data obtained from BRAIN-IT project; GP models used 19 dynamic predictors	AUROC: 0.872, acc: 0.774, sens: 0.816, spec: 0.752	30 min (starting 4 h after commencing data collection)
McNames, et al. (2013)	ICP, ABP	125 Hz	Signal down sampling, signal average subtraction, Blackman window segment multiplication, zero-padding to improve frequency resolution	Morphology analysis: FFT analysis of each segment	>20 mm Hg	Signal segments spanning 30 sec prior to the elevation compared with other 30-sec segments spanning 90–210 sec prior to the elevation; segments labeled consecutively	"ICP signal metrics may serve as precursors that characterize the transition zone prior to a rapid elevation and may enable prediction of these elevations up to 30 s ahead."	30 sec
Myers, et al. (2016)	ICP, MAP, ETCO_2_, SpO_2_, P_bt_O_2_	36-sec average	No	GP; logistic regression; AR-OR model	>20 mm Hg for >15 min	Noted to be unsuccessful in replicating Guiza, et al.'s work using a GP ML algorithm	AUROC 0.92 @ 15 min; AUROC 0.86 @ 30 min; AUROC 0.76 @ 6 h	15 and 30 min, 6 h
Guiza, et al. (2017)	ICP, ABP	1-min average	No	GP	>30 mm Hg for >10 min	External validation study applying Guiza, et al.'s GP algorithm to the retrospective analysis of data from 121 adult and 79 pediatric patients	Adult cohort: AUROC 0.90 (0.87–0.91); acc 86% (84%–88%), sens 70% (64%–76%), spec 90% (88%–92%)	30 min (starting 4 h after commencing data collection)
Hüser, et al. (2019)	ICP, ABP, ECG, SpO_2_, CPP	125 Hz waveform, 1 Hz vital signs	Filtering prior to computing non-overlapping 1-min block values calculated from high-frequency waveforms	L2-regularized logistic regression optimized using stochastic descent; single decision tree; gradient-boosting ensemble of decision trees; MLP + sigmoid activation	>20 mm Hg for >5 min	Data obtained from MIMIC-II waveform database	For an ICP event anytime in the next 8 h: PPV 0.36, recall/sens: 0.90	15, 30, 60, 90, 120, 240, 260, and 480 min
Carra, et al. (2020)	ICP, ABP	1-min average	No	GP	>30 mm Hg for >10 min	Follow-up validation study by Guiza, et al.'s team	AUROC 0.93, acc 88%, sens 83%, spec 91%	30 min (starting 4 ho after commencing data collection)
Smit (2020)	ICP	Frequency unavailable	ICP signal moving average filtered using 30 samples and transfer coefficients b = 0.0333 and a = 1; signal filtered forward and backward to eliminate phase shift; pulse averaging and normalization pre-processing	KSR; MOCAIP + classification tree; MOCAIP + LSTM; MOCAIP + CNN	>22 mm Hg for >5 min	First use of MOCAIP metrics in patients with TBI	KSR: sens 88%, spec 43%, acc 78%; LSTM: sens 97%, spec 66%, acc 70%; CNN: sens 47%, spec 60%, acc 58%	5, 10, 15, and 20 min
Schweingruber, et al. (2022)	ICP, ABP, SpO_2_, CPP, GCS, RASS, T, RR, HR, Vent. parameters, demographic, and lab data	1-h averaged values	Averaging	RNN with LSTM	Short critical phase: ICP >22 mm Hg during a 2 or less consecutive hour blocks; long critical phase: ICP >22 mm Hg during 2 or more consecutive hour blocks	80:20 train, test, and internal validation followed by external validation using MIMIC-III and eICU collaborative research databases; mixed cohort (SAH, TBI, CVA, etc); first tIH prediction algorithm able to be operationalized	Internal validation: AUROC (ICP-ICU test-set) = 0.98 ± 0.0008; external validation: AUROC: MIMIC-III = 0.965 ± 0.0010; eICU = 0.941 ± 0.0025; acc: MIMIC-III 90.7% (CI 90.1%–91.2%); eICU 87.1% (CI 86.6%–87.6%)	60 min

ABP, arterial blood pressure; acc, accuracy; ANN, artificial neural network; ANOVA, analysis of variance; AR, autoregression; ARV, average relative variance coefficient; AUROC, area under the receiver operating characteristic; CVA, cerebrovascular accident; CI, confidence interval; CNN, convoluted neural network; CPP, cerebral perfusion pressure; ECG, electrocardiogram; ETCO_2_, end-tidal carbon dioxide; FFT, fast Fourier transformation; GOS, Glasgow Coma Score; GP, Gaussian processes; HR, heart rate; ICP, intracranial pressure; KSR, kernel spectral regression; LR, logistic regression; LSTM, long short-term memory; MAP, mean arterial pressure; MD, mean difference; ML, machine learning; MLP, multi-layer perceptron; MOCAIP, morphological clustering and analysis of intracranial pressure; OR, ordinal regression; PPV, positive predictive value; RAP, correlation coefficient between ICP pulse amplitude and mean ICP; RASS, Richmond Agitation Sedation Scale; RNN, recurrent neural network; RR, respiratory rate; SAH, subarachnoid hemorrhage; sens, sensitivity; spec, specificity; SpO_2_, oxygen saturations; T, temperature; TBI, traumatic brain injury; tIH, traumatic intracranial hypertension; Vent., ventilator.

### ICP waveform morphology-guided tIH prediction

The application of ICP waveform morphological analysis to the task of predicting tIH events is consistent with the long-standing use of waveforms in clinical practice. ICP waveform morphology is well known to change with alterations in cerebral compliance and ICP.^19,21,61,62^ Thus, it is understandable that ICP waveform morphological analysis was an early approach used to predict tIH.

The first noted use of ICP waveform morphology analysis to detect ICP rises was published by Bray and associates in 1986.^63^ In this effort, discrete Fourier transformation of the ICP waveform was undertaken with the goal of developing a continuous ICP forecasting monitor.^63^ Using 100 Hz ICP wave data captured from 48 patients with sTBI, the group found that five pathophysiological states could be identified by analysing variations in the centroid of the high- and low-frequency components of the ICP waveform.^63^ In 2001, McNames and co-workers described their attempt at developing an ICP waveform morphology-based tIH prediction algorithm.^64^ In this effort, they divided 10- to 30-min blocks of wave data, which immediately preceded an ICP rise, into five segments.^64^ The group found that, in the transition from a stable ICP to an unstable ICP, the spectral power of the cardiac component of the ICP waveform reduced significantly prior to tIH.^64^ In 2010, Fan and colleagues retrospectively examined the rates of change in mean, SD, and variance of ICP on a 1-min basis for 30 consecutive minutes prior to a tIH event. They found that the slope of the ICP waveform increased in the minutes prior to tIH.^60^

### Morphological clustering and analysis of intracranial pressure

**FIG. 1.**
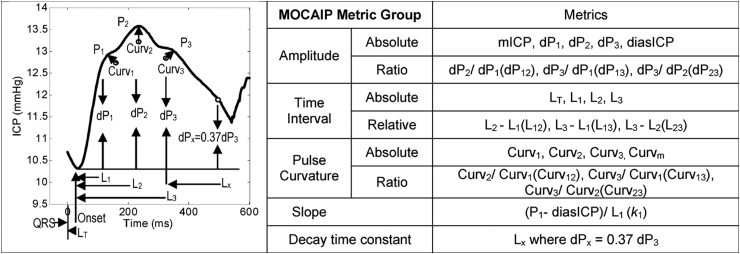
Illustration of MOCAIP metrics. ICP, intracranial pressure; MOCAIP, morphological clustering and analysis of intracranial pressure. Reprinted from Hu X, et al.^56^ Copyright 2010 by IEEE. Reprinted with permission.

A method of morphological analysis of individual ICP pulses was first presented by Hu and colleagues in 2009.^65^ The method developed identified and measured ICP sub-peaks, thereby allowing for detailed analysis of each ICP pulse using the group's morphological clustering and analysis of intracranial pressure (MOCAIP) algorithm (Fig. 1). To achieve this, the MOCAIP algorithm performs three tasks: ICP pulse recognition, ICP pulse sub-peak detection, and identification of ICP sub-peak labeling and measurement.^65^ Using this approach, the ICP pulse was analysed using 24 different MOCAIP metrics. Subsequently, MOCAIP analysis was combined with quadratic classification and the bootstrap method of statistical sampling to process ICP pulse data.^66^ Using this approach and the IH definition of an ICP rise of ≥20 mm Hg for >5 min, the group applied the MOCAIP approach to the task of IH prediction.^66^

In their first attempt, the MOCAIP method demonstrated 90% sensitivity and 75% specificity in predicting IH 20 min prior to an event.^66^ In a later study, the group identified an optimal subset of MOCAIP metrics using a differential evolution ML algorithm. Using this subset of MOCAIP metrics applied to retrospective data, IH episodes were differentiated from control segments at a specificity of 97% and sensitivity of 78% at a time horizon of 5 min prior to the ICP elevation.^56^ In a subsequent study, the group published results using the extremely randomized decision tree (Extra-Trees) ML algorithm to process MOCAIP data derived from a cohort of 30 patients at risk for IH. Applying the Extra-Trees ML algorithm to process the 13 best MOCAIP metrics for time horizons of 1, 3, and 6 min yielded an area under the receiver operating characteristic (AUROC) of 0.87, 0.78, 0.71; a sensitivity of 0.76, 0.62, 0.52; and a specificity of 0.72.^57^ It is noted that the MOCAIP algorithm was developed and validated using patient data from the University of California, Los Angeles Acute Hydrocephalus Center and did not involve any patients suffering from TBI.

Most recently, a master's thesis by Smit detailed attempts to use two ML approaches (a convolutional neural network [CNN] and a LSTM neural network) to classify IH and non-IH conditions using MOCAIP metrics.^28^ In this effort, which only included m-sTBI patient data, Smit noted that waves preceding tIH could be distinguished from control waves using MOCAIP metrics with a sensitivity of 89% and specificity of 71%.^28^ It was also noted that the classification of pre-IH wave based on MOCAIP at specific timings prior to an event was limited.^28^ The requirement for stable, patient-specific, high-resolution waveform data combined with the high computational requirements of morphological analysis has thus far precluded the clinical application of MOCAIP and other morphology-based approaches.

### tIH prediction algorithms using machine learning

The first use of ML to specifically predict tIH occurred in 2000 when Mariak and associates,^67^ in a continuation of earlier work by Swiercz and co-workers,^37,44^ described using an ANN algorithm to automatically assign an observed ICP waveform to one of four expert-determined risk classes (good, moderate, serious, and severe). Although no definition of the risk classes was provided, the ANN algorithm was consistent with expert scoring 70% of the time.^67^ In 2004 Shieh and colleagues described a modified RNN architecture that combined Elman architecture of the simple RNN structure through time (SRNNTT) and back propagation for the purposes of ICP prediction.^20^ Computer simulation demonstrated that SRNNTT combined with back propagation outperformed three other comparable network architectures (simple three-layer back propagation neural network, simple three-layer back propagation neural network through time, and Elman's recurrent neural network).^20^ However, data from only six patients were used to develop the model and no degree of clinical utility was demonstrated.^20^ Since these early efforts, more groups have subsequently developed novel ML-based tIH prediction algorithms.^26,58^

In 2013, using data collected in 2005, Güiza and associates built predictive models using multi-variate logistic regression and the ML technique known as a Gaussian process (GP).^59^ GP ML processing is based on the concept that any finite number of values from a collection of random variables from a known population will have a consistent Gaussian distribution.^68^ The GP algorithm developed by Guiza and associates identifies whether the distribution of a given number of vital sign measurements corresponds to a GP distribution of normal or high ICP values and provides an alert accordingly. Applying this technique to retrospective data, the developed GP ML algorithm had a sensitivity of 82% and a specificity of 75% for predicting an tIH event 30 min prior to the event. In this and all subsequent studies by Güiza and associates, a tIH event was classified as a rise in ICP ≥30 mm Hg that lasted for at least 10 min.^59,69,70^

In 2016, Myers and co-workers described their use of the autoregressive-ordinal regression (AR-OR) ML technique to approach the problem of tIH prediction.^26^ In autoregression ML, the algorithm uses present and previously observed correlations in a data set to predict future values. The addition of ordinal regression by Myers and co-workers served to dichotomize the algorithm output according to the study's tIH event definition of an ICP ≥20 mm Hg that lasted for 15 or more minutes.^26^ With the use of the AR-OR methodology, Myers and co-workers demonstrated the ability to predict an ICP event 30 min prior with an AUROC of 0.85. The group was noted to have also attempted to replicate the GP approach used by Güiza and associates without success.^26^ In 2017, Güiza and associates published validation results of the GP algorithm's performance against data from a different cohort of patients with m-sTBI, which confirmed that the model was robust among different ages of patients with m-sTBI.^69^

In 2021, Güiza and associates's group undertook validation testing using the high-resolution CENTER-TBI data set.^70^ In this study, the group's GP algorithm was demonstrated to have good intercenter robustness, with the model achieving an accuracy of 88%, a sensitivity of 83%, and a specificity of 91% in providing a 30-min forewarning of a tIH event.^70^ It is noted that the GP approach, although promising with retrospective data, is computationally intensive and requires 4 h of input data to allow it to make a prediction. Another more recent novel approach has been published by Hüser and colleagues in 2020 using data from the Multi-Parameter Intelligent Monitoring in Intensive Care II waveform database (MIMIC-II WFDB).^58^ In this study, the IH event was defined as five successive 1-min blocks with a mean ICP >20 mm Hg. The group used multiple ML approaches (multi-layer perceptron with sigmoid activation, single decision tree methodology, gradient-boosting ensemble of decision trees, and a regularized logistic regression modeling optimized using stochastic gradient descent) and the Shapley Additive exPlanations (SHAP) attribution technique to analyze the multi-scale descriptors of cerebral autoregulation indices, waveform morphology metrics, spectral energies, and statistical summaries.

With this approach, the group demonstrated that the addition of 125 Hz waveform data to numeric vital sign averages improved the prediction ability of the developed algorithm.^58^ With the inclusion of waveform data, Hüser and colleagues demonstrated the ability to predict IH events up to 8 h in advance with a precision of 0.442 ± 0.006 at 70% recall.^58^ Importantly, the group describes the recommended measures (recall and precision, which are known in clinical research as sensitivity and positive predictive value, respectively) that should be used when assessing tIH algorithm performance. Thus, although many articles describe an algorithm's AUROC, sensitivity, and/or specificity, for adequate comparison of algorithm performance, precision (positive predictive value) and balanced accuracy data are also necessary.

Most recently, Schweingruber and associates published details of an RNN-LSTM tIH prediction algorithm that can be operationalized for use in prospective clinical studies.^29^ The tIH definitions used for training the algorithm were termed short and long critical phases. A short critical phase was defined as any instance of ICP >22 mm Hg during 1 or 2 consecutive-hour blocks, whereas a long critical phase was defined as any instance of ICP >22 mm Hg during 2 or more consecutive-hour blocks.^29^ The time horizon of the algorithm was 1 h. The RNN-LSTM algorithm was trained using an 80%:20% train:test ratio of averaged vital sign wave data and a variety of other numeric data points. The source of the training data was a mixed cohort of ICP-monitored patients (TBI, subarachnoid hemorrhage, stroke, etc.). The trained algorithm subsequently underwent external validation testing using the MIMIC-III, eICU, and PhysioNet.org databases. The developed algorithm demonstrated good performance characteristics on the test set data, with an AUROC of 0.95 ± 0.0009 to predict the two targets within a 2-h horizon. On external validation testing using MIMIC-III data, the AUROC was 0.948 ± 0.0025 and using the eICU data set, 0.903 ± 0.0033.^29^

### Predictive factors

Features identified as predictors for an IH event vary according to the algorithms and input data used. Fan and co-workers, using statistical methods, found that a significant increase in the linear and quadratic slope of mean ICP occurred prior to a rise in ICP.^60^ Hu and colleagues noted that a 13-metric subset of the 26 MOCAIP metrics was optimal for predicting a tIH event (Fig. 1).^56^ Scalzo and associates found that the cardiac pulse amplitude of the ICP wave form (Cpa), average arterial blood pressure (ABP), ICP mean-amplitude correlation (pma), and ICP amplitude in the 30 sec prior to a tIH event were predictive factors for a tIH event. In the study by Scalzo and associates, a tIH event was defined as ICP ≥20 mm Hg lasting ≥5 min.^57^ The GP algorithm developed by Güiza and colleagues relies on ICU length of stay (LOS) at prediction time +73 dynamic predictors.^59,69,70^ The dynamic predictors include 51 pertaining to ICP (including absolute values, signal variability, and frequency-domain coefficients), 14 to CPP (including absolute values for the first hour and frequency-domain coefficients), 5 to ICP-MAP correlations, and 3 to the most recent MAP variability.^59^

The AR-OR algorithm developed by Myers and co-workers mostly relied on the most recent algorithm output, the last two measurements of ICP, and the time since last crisis.^26^ Similar to earlier work by Scalzo and associates, McNames and colleagues noted that the Cpa and the ρma were consistently lower in the 30-sec segment at the leading edge of an ICP elevation as compared with segments 1.5 to 3.5 min prior to the edge.^64^ McNames's team also found that heart rate, as measured by the cardiac component peak frequency (Cpf) and the average ABP, was consistently higher in the same segment.^64^

Major predictors used by the algorithms of Hüser and co-workers included the pulse diastolic, spectral energy (0–1 Hz band), slow wave index, and pulse slope of the weighted ICP (wICP).^58^ In addition, the average ICP was also used by the algorithm.^58^ The algorithm of Smit and associates focussed on five MOCAIP absolute amplitudes (mICP, dP_1_, dP_2_, dP_3_, and diasICP) in the cohort of patients with TBI studied. For the algorithm developed by Schweingruber and colleagues,^29^ the two top features from each domain used to predict a short phase event were vital signs: MAP and mean airway pressure; descriptive: gender and weight; blood gas analysis: pCO_2_ and potassium; medication: opioid and catecholamine; and laboratory: erythrocytes and eosinophils. For prediction of a long phase event, the top two features for each domain were vital signs: MAP and CPP; descriptive: weight and gender; blood gas analysis: HCO_3_ and potassium; medication: opioid and benzodiazepine; and laboratory: thrombocytes and erythrocytes. It is noted that the Schweingruber and colleagues integrated gradient-enhanced saliency mapping was undertaken in the temporal domain rather than in the frequency domain.^29^

Caution must be used when interpreting factors used by an algorithm to make a tIH prediction. If only ICP data are used for algorithm training, it follows that the trained algorithm will be reliant on ICP data to make a prediction. Further variation arises from the type of algorithm used. Using identical training data and target outcomes, an autoregression algorithm will rely on different feature data in comparison with an LSTM algorithm to achieve the same designated task. As determinations about capture frequencies, types of data, algorithm architectures, target event definitions, horizons, and other such parameters are made, feature data are accordingly influenced. Algorithm feature data need to be interpreted in the context of these factors. Using large, high-resolution data sets and semi-supervised ML techniques may yield less biased results. In supervised ML training, the outcome is designated by humans and available data are presented to the computer. In unsupervised training, target determination decisions are made by the algorithm. Drawbacks of unsupervised training methods may include large computational processing requirements and a tendency for overfitting. With unsupervised training there is a risk that models exceeding human interpretability may be generated.^71^

Determining feature data used by deep learning algorithms, such as LSTMs, is a task that requires special consideration. Deep learning algorithm feature identification is achieved using integrated gradient (IG) processing.^72,73^ IG processing, a sub-type of saliency mapping, identifies key features and anomalies and attributes feature importance.^72,73^ In IG processing, data within the algorithm's prediction horizon are analyzed in either the spatial (if image data), temporal, or frequency domain to identify key features and anomalies.^74–77^ To explain causal association, IG processing generates a series of two-dimensional images in which the pixel color attributes the importance of the data for the prediction task. For IG processing of time-series data, frequency (not temporal) domain analysis using wavelet or Fourier's fast transformation (FFT) is the recommended approach.^74–77^ This is to both improve interpretability and to account for the possibility of a wandering baseline. In frequency domain IG processing, following FFT or wavelet data transformation, overlapping windows are used to analyse the regions preceding a tIH algorithm prediction.

### Technical barriers to the clinical implementation of tIH prediction algorithms

Over the past 40 years, numerous groups have used a variety of approaches to develop algorithms to warn clinicians of an impending IH event. Although, until the work by Schweingruber and colleagues, none of these efforts have been successful in developing an operationalizable tIH prediction algorithm, the body of work performed indicates that there are several neurophysiological signals that can provide a warning of an impending IH event. Thus, tIH prediction is possible and the challenge of real-time tIH prediction for bedside usage is technical in nature. For several of the distinct ML algorithm architectures, such as gradient boosting, tree-based methods, autoregressive approaches, GPs, and a variety of linear and non-linear regression algorithms, the amount of data preparation and curation required, the computational demands of the algorithm architecture, the requirement for large amounts of data prior to first prediction, and/or the requirement for artefact-free source data all present major barriers to operationalization.^68,78^

A variety of recurrent ML algorithms have also been used for both tIH prediction and ICP forecasting with varying degrees of success.^29,37,44,49,50,67,79^ Recurrent algorithms, which include RNNs, LSTMs, and other deep neural networks, are a family of algorithms designed to make predictions from temporal dynamic evolution of the input features.^80,81^ Temporal dynamics is the concept that driver–response relationships are not always constant through time, but rather are conditioned to varying degrees by both recent and remote past events.^82^ Temporal dynamic systems are inherently complex.

In ML, accounting for temporal dynamics is achieved by using recurrence equations, which are equations wherein the output is a function of multiple smaller input data units that include the output from recent prior calculations.^80,81^ In operation, the algorithm output at various timesteps is fed back into the same algorithm to inform the next series of calculations. This property of recurrence results in an internal memory for the family of algorithms, making them ideal for time-series data processing.^80,81^ Probability generation based on both input data and prior output calculations is the basis for the adaptation of recurrent algorithms to the task of tIH prediction. For this, algorithm tasking is modified so that the output parameter is the probability of a given target ICP sequence (i.e., ICP ≥25 for 10 or more minutes) occurring with a given input sequence. Accordingly, the prediction of a tIH episode by a recurrent ML algorithm occurs when, in response to an input sequence, the probability of an output of interest (i.e., a tIH event) rises above a predetermined threshold. It is of little surprise that the first operational tIH prediction system is a recurrent algorithm and further generations of tIH prediction technology will undoubtedly use both more advanced recurrent algorithms and higher density data sources. Integration of waveform data, as opposed to averaged data, for both training and operation of the latest generation of memory-enabled recurrent algorithms is a logical next step.^49,50,58,83^

One major technical barrier with ICP forecasting and/or tIH prediction with RNN ML algorithms relates to the sequential processing approach commonly used by the algorithm family.^84^ Sequential processing, wherein data temporality determines the hierarchical order of analysis,^85^ has several inherent drawbacks. First, it results in bottlenecks, which impede processing speeds and increase computational demands.^86,87^ Second, it often has demanding training requirements in terms of data, time, and computational requirements.^87^ Finally, it suffers from the problem of recency weighting. In recency weighting, more recent data are processed first and are inherently weighted higher than more temporally distant data. Thus, for analysis of time scale data such as continuous vital sign wave monitoring, more remote data may be inadvertently downweighted or ignored entirely (known as the vanishing sequence phenomenon).^86^ The addition of LSTM processing to RNN algorithms, which acts to store and re-present historical data segments to the algorithm at arbitrary intervals, helps to overcome some issues relating to recency weighting. However, the addition of LSTM processing does not fully resolve recency weighting and results in both slower processing speeds and an increase in computational demands.

## Algorithm Training

Training a ML algorithm begins with specifying the algorithm's objective or purpose. Following this, training data, algorithm architecture(s), and targets for training are identified. The training target must be represented in the training data. The algorithm training data source directly impacts any subsequent effort of operationalization (see section “Data capture and transmission” below) and must be considered. The amount of data required varies according to the algorithm used, training strategy, target event frequency, and type of data available. Insufficient data will result in overfitting of the trained algorithm. Excessive amounts of training data results in the regression to the mean phenomenon, which may result in failure to identify a predictive pattern.

The prediction time horizon is a balance between clinical utility and alarm fatigue. Too short a horizon will result in poor clinical utility, whereas too long a horizon will increase alarm fatigue and reduce algorithm accuracy. Once training data are identified they are divided into three unequal portions—one for training, another for testing, and the third for validation. This practice is known as the train:test:validation split rule and typically results in ratios of 60:20:20, 70:15:15, or 80:10:10 depending on the available data.^88–90^ The data are then pre-processed and standardized if required. Standardization of data capture obviates this requirement and preserves both data and computational resources while improving algorithm performance characteristics. However, the use of standardized training data may reduce the resiliency, in terms of the ability to process a variety of capture frequencies, of any developed algorithm.

Training an RNN involves tuning an algorithm's hidden layer nodes' weightings and biases to adjust algorithm output until the algorithm reliably and accurately achieves the outcome target of interest. Once trained, the algorithm nodes are fixed. Drift monitoring and retraining algorithms can be embedded to continually tune the hidden layer nodes to adapt to data variance that arises from the evolution of medical practice.

## Operationalization of tIH Algorithms

A tIH prediction algorithm must respond in a timely, reliable, and intelligible manner to be of clinical use. Although the development of a tIH prediction algorithm is a challenge, once achieved there are numerous additional technical barriers to be overcome to operationalize an algorithm. It is noted that although several dozen tIH prediction and ICP forecasting algorithms have been developed, up until very recently, none have been operationalized. This is consistent with the current state of artificial intelligence systems in the ICU environment. In a 2021 systematic review of the use of artificial intelligence in the ICU, it was noted that 96.4% of studies involved only retrospective testing of artificial intelligence algorithms.^91^ Eighteen of the 494 studies included in the analysis were prospectively evaluated in the clinical setting and only one was subjected to randomized controlled trial (RCT) evaluation.^91^

Streaming patient data to a tIH algorithm for processing in real time is not straightforward and requires the development of scalable data infrastructure. Operationalizing tIH algorithms involves establishing a multi-step data pipeline. The steps can be divided into four stages: 1) data capture and transmission; 2) data pre-processing; 3) tIH algorithm processing; and 4) prediction reporting/user interface. Building an operational tIH prediction system, or any system of this nature, encompasses nearly the entire remit of data science. Other related disciplines or concepts that may be required include embedded system engineering, devOPs (a set of practices that combine software development and IT operations), and cybersecurity. As noted by Allen in 1983, the endeavor “demands close co-operation between clinician and engineer (data scientist).”^41^ It is important to recognize that any data pipeline developed requires ongoing maintenance as the individual software applications used in the creation of the pipeline are periodically updated, retired, and/or replaced by newer applications. Pipeline sub-application updates often require re-programming and/or adjustment of the pipeline operating parameters and may also require re-establishment of pipeline data security measures.

### Data capture and transmission

tIH prediction algorithms require sufficient available data to provide timely predictions. Accordingly, operationalization of any tIH algorithm is dependent on the usual location of the data (Table 3). Algorithms reliant on access to a hospital database face privacy regulation, which varies between jurisdictions. In addition, the capture of demographic, laboratory, and/or medication data is variable in both timing and process across jurisdictions. Algorithms reliant on hospital network data are thus prone to being restricted to operating within health service networks and algorithm predictions may be delayed depending on data availability. One possible solution to regulatory barriers that limit the transmission of patient data are federated networks.^92^ In these networks, data are processed by algorithms on the hospital network and results, not patient data, are sent to an external network for further processing or analysis.^92–94^

**Table 3.** Overview of Potential Data Sources for Operationalization of TBI Machine Learning Algorithms

Data	Data source	Frequency	Average file size per patient/episode
Pathology, medical history, medication, ICD code, procedural and/or operational data, etc.	Patient administration systems, theater management systems, electronic health records, clinical information systems, etc.	<<1 Hz	<1 MB
Vital sign waveform data (ICP, ABP, ECG, CPP, etc.)	Bedside monitor	60–300 Hz	60 MB–1 GB
Vital sign numeric data (temperature, S_P_O_2_, HR, RR, NIBP, P_bt_O_2_, multi-modal data, etc.)	Bedside monitor, electronic health records, clinical information systems, wearable devices	∼1 Hz	10–30 MB
MRI/CT scan	DICOM server	<<1 Hz	1 GB–5 GB

ABP, arterial blood pressure; CPP, cerebral perfusion pressure; CT, computerized tomography; DICOM, digital imaging and communications in medicine; ECG, electrocardiogram; HR, heart rate; ICD, International Statistical Classification of Diseases and Related Health Problems; ICP, intracranial pressure; MRI, magnetic resonance imaging; NIBP, non-invasive blood pressure; P_bt_O_2_, brain tissue oxygenation; RR, respiratory rate; SpO_2_, oxygen saturation.

Swarm learning is another potential solution.^95^ In swarm learning, deep learning algorithm processing parameters (hidden layer nodal weights and biases), not the processed data, are securely shared between sites. This allows swarming algorithms to learn from the experience of other sites and improve performance while keeping patient data secure at the local site.^95^ Both swarm learning and federated networks require extensive data science expertise and resources to establish and maintain. An alternative approach is to stream non-identifiable patient data prior to it entering a hospital network. This is most possible with patient monitoring equipment and radiology systems and has the advantage of being deployable, standardizable, and able to provide high-density data in a timely manner. Whichever approach is used, tIH algorithm operationalization requires the data to be packaged, encrypted, compressed, pre-processed (if required), and subsequently securely transmitted to the algorithm server.

### Data pre-processing

Data pre-processing involves preparing the data for processing by the tIH prediction algorithm and is used to optimize tIH algorithm prediction performance.^96,97^ Pre-processing varies according to both data and algorithm requirements and can be broadly categorized into the following steps: data cleaning, integration, transformation, and reduction.^97^ Data cleaning includes outlier removal, interpolation of missing values, noise reduction, and duplicate record removal, etc. Data integration is the process of reorganizing data from multiple data sources into a single database. Data transformation converts the data into a format suitable for algorithm processing. Finally, data reduction refers to the removal of duplicate or extraneous records if required.^97^

Numeric data pre-processing can include data standardization, logarithmic transformation, binning, outlier removal, interpolation of missing values, and dimensionality reduction.^98^ Wave data pre-processing may include artefact removal, waveform clipping, waveform alignment, outlier removal, median filtration, wavelet transformation, and spatio-temporal data imputation.^99^ Radiological image data pre-processing may include white balance adjustment, tone and gain modulation, noise reduction and sharpening, image fragmentation, and color space transformation.^100^ Pre-processing can occur at any stage up until data reach the tIH algorithm(s) (i.e., before or after data transmission to the tIH algorithm host server), depending on the pre-processing computational requirements and the available streaming hardware. For an operational ML algorithm data pipeline, pre-processing systems must be automated and able to rapidly process streamed data (i.e., within tens of milliseconds to seconds).

### tIH prediction algorithm operation

Upon reaching the tIH algorithm, the pre-processed data undergo processing by the tIH algorithm. To facilitate this the computational requirements of algorithm operation require consideration. Processing capabilities are measured in terms of random access memory (RAM) and computational power (floating operations per second [FLOPS]). As data for an operational tIH algorithm are transmitted in batches every few seconds or minutes, operational processing requirements of even large ML algorithms are less compared with processing the same data retrospectively (i.e., processing an entire patient admission at once). Compute resources can be server- or cloud-based and require sufficient memory and processing power to simultaneously operate the number of algorithms required and to store the captured data. Cloud-based systems have the advantage of being scalable. This allows for expansion and contraction of processing power and/or memory capacity according to demand. In addition, cloud systems allow for techniques such as auto-calibration, swarm learning,^95^ and/or co-location to multiple jurisdictions. With co-location, cloud systems are an equitable and efficient option. This option is equitable as it allows sites that may lack the requisite IT infrastructure needed to develop artificial intelligence systems in their local environment (i.e., data science expertise, high-performance computers, and/or cloud networking) access to the technology. And it is an efficient option as co-location avoids resource duplication.

### Prediction reporting/User interface

Following algorithm processing, the outcome from the tIH prediction algorithm analysis must be made available to clinical staff for consideration and response if warranted. The tIH algorithm output must be timely, reliable, accessible, and intelligible. Without a clinical response to an alarm, the system will not impact patient care. tIH prediction algorithms should be calibrated to avoid alarm fatigue, a well-known problem in the ICU environment.^101–103^ Options for dissemination of tIH algorithm alerts include directly to bedside computers or monitoring equipment, via short messaging service (SMS) text messaging, via web-based portals, and/or via other secure internal messaging systems such as Microsoft Teams.

Once developed and operationalized, algorithms need to demonstrate acceptable performance during observational testing. Pilot studies examining algorithm-guided protocol feasibility are required. Subsequent Phase 2 trials are needed to demonstrate the ability of tIH prediction algorithm-guided treatment to show benefit in terms of a surrogate marker, such as ICP burden and/or number of tIH events. Only after these and multiple potential other studies have been completed will a larger Phase 3 RCT, designed to assess the impact of algorithm-guided treatment on one or more patient-centred outcomes, be warranted. Guidelines for the design and reporting of clinical trials involving artificial intelligence technology are provided by the Standard Protocol Items: Recommendations for Interventional Trials – Artificial Intelligence (SPIRIT-AI)^104^ and Consolidated Standards of Reporting Trials – Artificial Intelligence (CONSORT-AI)^105^ documents, respectively.

### Statistical analysis

With the operationalization of tIH prediction technology, consideration of the statistical underpinnings of prediction artificial intelligence technology is necessary. Algorithms can be classified according to the methodology used: statistical methods, ML, and deep learning (Table 4). The mechanistic differences between deep learning and statistical approaches lead to a different approach in assessment of respective algorithm performance. Statistical approaches generate population inferences from a sample.^106^ Deep learning algorithms typically use neural networks to identify predictive patterns in data.

**Table 4.** Overview of the Statistical Assessment of tIH Prediction Algorithms

	Statistics	Machine learning	Deep learning
Method	Interpolation	Variable	Pattern identification
Performance assessment tools	Overall performance: Brier score or R^2^ statistic Discrimination: Balanced accuracy, sensitivity (recall), specificity, positive predictive value (precision), negative predictive value and/or goodness of fit modeling Calibration: Goodness of fit modeling	Variable: Dependent on algorithm methodology. If extension of statistical model, then use of statistical performance assessment is appropriate.	Discrimination: Balanced accuracy, sensitivity, specificity, positive predictive value, negative predictive value

For statistical and ML prediction algorithms that are extensions of statistical models, the outcome is binary and expressed as a probabilistic (or stochastic) prediction. Probabilistic predictions are absolute (as opposed to relative) risks. For the assessment of tIH (or other predictive algorithms) that are based on statistical or statistically derived ML models, assessment of the overall algorithm performance, calibration, and discrimination and is appropriate.

Assessment of overall algorithm performance is performed using the Brier score and/or the R^2^ statistic, both of which provide values between 0 and 1 (or 0% and 100%). Both measures assess variance between predicted and observed outcomes. A Brier score of 0 indicates perfect prediction accuracy, whereas a score of 1 indicates perfect prediction inaccuracy. Conversely, an R^2^ statistic of 1 indicates perfect algorithm accuracy, whereas an R^2^ statistic of 0 indicates perfect algorithm inaccuracy. Calibration is assessed using goodness-of-fit modeling.

**FIG. 2.**
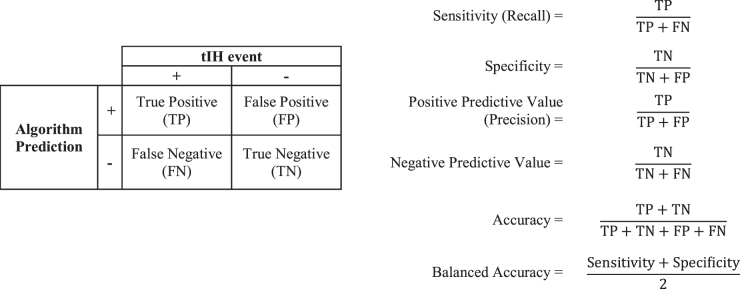
Predictive performance parameters. Calculation of sensitivity, specificity, positive predictive value, negative predictive value, accuracy, and balanced accuracy.

Algorithm discrimination is assessed by measurement of predictive performance parameters and/or goodness-of-fit modeling. Predictive performance parameters include sensitivity, specificity, balanced accuracy, positive and negative predictive values, and the AUROC. Due to the large number of true negative predictions generated by tIH prediction algorithms, balanced accuracy is used instead of accuracy (Fig. 2).

Goodness-of-fit modeling is an alternative and often complementary method of algorithm discrimination assessment. In these models, better performance is indicated by smaller differences between predicted and actual outcome. The advantage of goodness-of-fit modeling is that it is often assessable using the same data set, whereas predictive performance parameter determination frequently requires new data or cross-validation.^107^

Complicating algorithm discriminative assessment is the dynamic nature of deep learning tIH algorithms. Deep learning algorithms are identifiable by their frequent use of neural networks, the capacity to self-correct. This latter capability arises from deep learning algorithms' relative immunity to feature engineering. This differs from ML algorithms and statistical models, which require human input to make corrections. In addition, extensive feature engineering is often required to develop such algorithms. In deep learning algorithms based on recurrent networks, memory of relevant events or patterns is generated during processing. A recurrent network's hidden state (a term used to describe an algorithm's operational memory) is dynamically adjusted during operation in response both to changing input sequences and past features and predictions. This results in modulation of algorithm performance and consequent individualization of processing. Thus, with access to a larger patient context, a deep learning tIH algorithm's performance continuously improves (also known as “continuously adapting” and/or “continuously evolving”) for each patient as it self-adjusts during operation.^105^ To account for this and to support clinical interpretation, deep learning and/or pattern-based ML tIH algorithms often display performance data in real time.

For deep learning approaches that involve pattern identification, the required determination is how well the identified pattern(s) can predict an outcome in a data set. The application of statistical regression models is not appropriate as there is no regression or inferencing occurring. Accordingly, discrimination assessment in terms of balanced accuracy, sensitivity, specificity, and positive and negative predictive values is the standard used in the assessment of deep learning predictive algorithms. Assessment of ML algorithms is variable and is dependent upon the nature of the processing algorithm. Some forms of ML, such as GP models, are extensions of statistical models. In these cases, the application of statistical regression analyses to assess performance is appropriate. With other forms of ML, particularly more advanced recurrent algorithms such as LSTMs, adoption of the standard used for deep learning is appropriate.

## Risks of tIH Prediction Technology

With any new clinical technology, consideration of the risk presented by its adoption is warranted. For tIH prediction algorithms, in the event of a false-negative alarm and/or the loss of system functionality, clinical management reverts to standard care. As such, these events present negligible risk compared with current practice. The main risk presented by this technology is thus false-positive alarms, which can result in both alarm fatigue and/or overtreatment of patients.^102,103^ The degree to which this is a problem is dependent on the clinical context and the performance characteristics of the algorithm. Pragmatically, responding to a tIH algorithm alarm would entail a patient review including assessment of the patient's autoregulatory status. Subsequent actions may include preparing for a rise in ICP (e.g., having drugs available at the bedside, deciding on course of treatment in event of an ICP rise, etc.), optimization of clinical parameters (e.g., adjusting ventilation, optimizing sedation, raising CPP toward upper limit of target range if cerebral autoregulation is intact), and/or delaying potentially ICP-aggravating procedures (e.g., suctioning or rolling).^29,108^ In high-risk patients, initiating investigations (brain computed tomography [CT]) and/or tier 1 ICP management prior to an ICP rise may be warranted.

Although there is little data on the effect of further lowering safe ICP levels (i.e., from 14 mm Hg to 6 mm Hg) observational data suggest that doing so is not harmful and may be beneficial as it lowers the overall ICP burden.^31^ In the setting of tier 1 treatment of raised ICP, the specific risks are related to the treatment selected.^8,9^ The risks associated with the intra-hospital transfer of critically ill patients are well described.^109^ However, the transport of a patient to a CT scanner or operating room during a tIH episode is, although common, of particularly high risk.

It is important to recognize that there is no RCT evidence that treatment of tIH improves patient outcomes. In some cases, treatments such as hyperventilation have been shown to cause harm.^110^ In addition, untargeted prophylactic treatment of IH has also been shown to not improve outcomes and/or cause harm, albeit with interventions that are now considered tier 3 measures and are used less frequently.^110^ In all studies, the investigative treatments were either administered after IH had become established or blindly to patients prophylactically. In studies examining the treatment of tIH, time to intervention administration often occurred after several hours of elevated ICP.^12,14^ Whether or not a targeted preemptive strategy will improve outcomes remains unknown. Only through the rigorous clinical evaluation of tIH prediction technology will it become possible to determine whether preemptive treatment of tIH is of no use, is of benefit, or is harmful.

In support of the safety of preemptive treatment are studies by Pakkanen and colleagues^111^, Huijben and associates,^112^ and Robba and co-workers.^11^ Their investigations found that higher therapeutic intervention levels (TIL) and/or earlier availability of expert attention were associated with improved outcomes in patients with TBI. None of these observational studies suggest harm from exposure to a higher TIL. There is also a body of evidence associating ICP burden with poor outcomes.^5,7,26,33,35^ Of note, Myers and colleagues demonstrated that time spent in crisis inversely correlated with 3- and 6-month Glasgow Outcome Score (GOS).^26^ Finally, the most common factor used to predict a future ICP rise is the historical ICP value.^26,28,29,57–59^ This supports the concept that IH is both a cause and consequence of secondary brain injury.

With the impending availability of tIH prediction technology there is an opportunity to ensure that rigorous clinical testing occurs prior to clinical implementation. Favorable, adequately powered, Phase 3 multi-center RCTs delineating tIH algorithm efficacy in terms of patient outcome and/or improved operational performance should be the standard for implementation.

## Key Points

- tIH prediction algorithms have begun clinical testing.
- Operationalization of a tIH algorithm involves establishment of a data pipeline to transfer data from the patient to the tIH prediction algorithm, and subsequent transmission of algorithm predictions to the user.
- Whether or not tIH prediction algorithms will be of benefit in terms of improving patient outcomes remains to be determined.

## Conclusions and Future Research

ML algorithms are a developing part of medical practice. As such, the development of operational tIH prediction algorithms will represent an early milestone in the application of ML and data science to medicine. With improvements in methodology and technology, the main barrier to implementation and clinical integration now lies not with algorithm development but rather with the operationalization of the technology. Once developed and operationalized, much work will need to be undertaken to evaluate and optimize the new technology. Much of this will inevitably occur in a heuristic manner; however, a systematic collaborative approach between technically capable centers will accelerate the development process.

For any developed operational tIH prediction algorithm, it remains to be determined if preemptive tIH management can reduce the ICP burden and/or number of tIH events experienced by patients. If established, determination of whether preemptive management of tIH results in either improved patient outcomes and/or care is required. As any benefit of an early warning stems from the resulting intervention, determination of the optimal clinical response to a tIH algorithm alarm is required. Given the dynamic and heterogenous nature of TBI, the optimal clinical response is likely to vary both between patients and over time. Optimal tIH target(s), horizon timing(s), training data, data capture frequencies, and data pre-processing systems also remain to be determined. Potential alternative targets include different ICP thresholds and/or targeting intracranial compartment syndrome (i.e., the combination of tIH and CPP values below the lower limit of cerebral autoregulation).^19^

Other operative elements that affect tIH algorithm delivery and performance require consideration. Decisions regarding algorithm delivery systems (i.e., cloud-based vs. hospital server-based vs. hybrid infrastructures) will have implications on future health artificial intelligence technology development, resource utilization, and equitable access. With the ability to operate multiple algorithms simultaneously, operating a combination of tIH prediction algorithms with different target thresholds and/or in combination with ICP forecasting algorithms may be an effective strategy.

Other considerations include: 1) integration of other modes of data, such as demographic, CT imaging, and/or medication data to further enhance algorithm performance; 2) pre-processing system configuration; 3) understanding and optimizing human interface with the system; 4) assessment of the utility of multi-modal data integration, such P_bt_O_2_, lactate pyruvate levels, and/or spreading depolarization waves; 5) adoption of alternative thresholds; 6) assessment of the generalizability of any algorithm(s) in patient, pathophysiological, and/or geographic terms (i.e., algorithm performance in pediatric patients? in aneurysmal subarachnoid hemorrhage, acute hydrocephalus, and/or other causes of IH? in different geographic populations?); and 7) calibration mechanisms to maintain algorithm performance in the face of continuous clinical practice evolution. To date, technical barriers have meant that it has not been possible to directly address the above issues. However, tIH prediction algorithms currently undergoing clinical testing may begin to answer some of these questions.^29^ As clinical integration of the technology progresses, it is appropriate to remember that “treatments with a strong rationale should be tested in methodologically rigorous multicentre RCTs, and not implemented on the basis of biological plausibility, low-quality studies, and presumed safety.”^113^
